# Net-FLICS: fast quantitative wide-field fluorescence lifetime imaging with compressed sensing – a deep learning approach

**DOI:** 10.1038/s41377-019-0138-x

**Published:** 2019-03-06

**Authors:** Ruoyang Yao, Marien Ochoa, Pingkun Yan, Xavier Intes

**Affiliations:** Department of Biomedical Engineering, Rensselaer Polytechnic Institute, Troy, NY 12180 USA

**Keywords:** Biophotonics, Imaging and sensing

## Abstract

Macroscopic fluorescence lifetime imaging (MFLI) via compressed sensed (CS) measurements enables efficient and accurate quantification of molecular interactions in vivo over a large field of view (FOV). However, the current data-processing workflow is slow, complex and performs poorly under photon-starved conditions. In this paper, we propose Net-FLICS, a novel image reconstruction method based on a convolutional neural network (CNN), to directly reconstruct the intensity and lifetime images from raw time-resolved CS data. By carefully designing a large simulated dataset, Net-FLICS is successfully trained and achieves outstanding reconstruction performance on both in vitro and in vivo experimental data and even superior results at low photon count levels for lifetime quantification.

Lifetime, which is an intrinsic property of fluorescent molecules, describes the time that a fluorophore spends in the excited state before returning to the ground state. Fluorescence lifetime imaging (FLI) provides unique additional information to intensity imaging with the advantages of being independent of the fluorophore concentration (before quenching) and minimal susceptibility to the changes in optical properties. Fluorescence lifetime imaging microscopy (FLIM) is widely applied to increase the multiplexing power, sense changes in microenvironment (pH, pO_2_, etc.)^1^, and monitor molecular interactions such as performed via Förster resonance energy transfer (FRET) studies. Coupled with multispectral or hyperspectral imaging, FLIM can unveil enriched information in the lifetime and intensity spectra^2^. Recently, we reported the development of a hyperspectral time-resolved wide-field system for macroscopic fluorescence lifetime imaging (MFLI)^3^. The system supports structured light illumination and single-pixel detection over an 8 × 6 cm^2^ field of view (FOV) via two digital micromirror devices (DMDs) and hyperspectral (80 nm range in 16 wavelength channels) data acquisition via a Photomultiplier Tube based Time-Correlated Single Photon Counting (PMT-TCSPC) spectrophotometer, which enables efficient whole-body FLI of live subjects. With time-resolved CS signals ***S*** collected with patterns ***P***, both intensity image ***I***^***A***^ and lifetime image ***I***^***τ***^ can be reconstructed.

However, the data-processing workflow necessesary to generate the spatially resolved quantitative FLI images can be time-consuming, susceptible to noise and requires user inputs to define the key parameters of the core iterative procedures. More precisely, the classical workflow recovers ***I***^***A***^ and ***I***^***τ***^ from three steps: (1) Solve the inverse problems ***PI***^***A***^(*t*) = ***S***(*t*) with CS-based^4^ solvers for each time gate *t*, where ***P*** is the sensitivity matrix formed by the pattern weights. (2) Retrieve lifetime values *τ*_*i,j*_ from the time point spread function (TPSF) curve ***C***_*i,j*_ with the least squares method (LSM)-based fitting for every pixel of interest, where *i* and *j* are the row and column indices of the image. (3) Repeat (1)–(2) for each wavelength channel. Then, the reconstruction accuracy of the FLI output images can depend on the selected regularization and (bi)-exponential fitting parameters (e.g., in tail fitting). Hence, there is great interest in developing a faster and more robust imaging workflow to translate CS-based MFLI to bed-side applications and for wide acceptance by non-specialists.

In this regard, recent developments in deep learning (DL) provide new avenues for image formation and processing. Indeed, DL has led major advances in various fields such as computer vision (CV) and natural language processing (NLP)^5^. In particular, convolutional neural networks (CNN) with convolutional layers for multilevel feature extraction are successfully used for image processing^6^, object recognition^7^, image super resolution^8^, and even reconstruction^9–11^. Thus, they are well suited for medical imaging tasks, such as tissue lesion detection and segmentation^12^, label-free cell classification^13^, and super-resolution microscopy^14^. In this letter, we report the design and validation of a deep CNN named Net-FLICS (fluorescence lifetime imaging with compressed sensing), which enables direct DL-based fluorescence intensity and lifetime imaging from time-resolved single-pixel datasets for the first time. In addition to significantly shorter data-processing time compared to the classical workflow (referred to as TVRecon) based on the TVAL3^15^ inverse solver and LSM fitting, Net-FLICS demonstrates better quantitative accuracy for common MFLI applications. The network was entirely trained on a model-generated simulation dataset, but successfully validated on in vitro and in vivo experimental datasets in the challenging case of Near-infrared (NIR) FLI, which is typically characterized by sub-nanosecond lifetimes.

The architecture of Net-FLICS shown in Fig. 1a is inspired by two network designs: ReconNet^9^ and Residual Network (ResNet)^16^. ReconNet can reconstruct intensity images from random single-pixel measurements. Composed of one fully connected layer and six convolutional layers, it is 2–3 magnitudes faster and more robust against noise than the state-of-the-art CS reconstruction algorithms. ResNet, which is characterized by a “skip connection” that bypasses nonlinear transformations, was used to address possible non-converging issues during the training of Net-FLICS^16^. It works by converting direct mapping to residual mapping and enables training for extremely deep neural networks. Net-FLICS takes an array of size 256 × 512 as the input, which represents 512 CS measurements with 256 time gates, and outputs an intensity image and a lifetime image, both of which have sizes of 32 × 32, as the reconstruction prediction. Net-FLICS contains three main segments: (1) A shared segment that recovers the sparsity information from CS measurements, which is similar to the first step in TVRecon^3^. The output from the first segment represents the temporal point spread functions (TPSFs) of each pixel; (2) An intensity reconstruction segment with one ResBlock and one ReconBlock; (3) A lifetime reconstruction segment with a 1D convolutional layer, two ResBlocks and two ReconBlocks. Further details regarding the input data, number of kernels, kernel sizes, activation function and batch normalization (BN) can be found in Part 1 of supplementary materials.

A large and comprehensive training dataset is critical for the performance of any deep CNN. However, since our hyperspectral wide-field platform was not introduced until very recently, there is insufficient experimental data. Thus, we designed a data generator, which mimics the process of compressed-sensing-based fluorescence lifetime imaging. The EMNIST dataset^17^, which contains ~7e5 28 × 28 grayscale images that represent digits or letters, was used as the raw data. From each grayscale image, an intensity image and a lifetime image were generated in the commonly seen range, i.e., 25–1600 photon counts and 0.3–1.5 ns for each pixel. For data augmentation purpose, each letter/digit might be rotated, and multiple images can be combined. With the intensity, lifetime, and time step of the imaging system, we obtained a decay curve for each pixel, which was then convoluted with the instrumental response function (IRF) to obtain a TPSF. Single-pixel measurements were calculated as the weighted sum of TPSFs from all pixels in a Hadamard pattern,  obtained by reshaping rows of a 1024×1024 Hadamard matrix. Finally, the time-resolved single-pixel data were added with Poisson noise, and a subset was selected as the input for Net-FLICS. A more complete description of the proposed data generator is provided in Part 2 of supplementary materials.

**Fig. 1:**
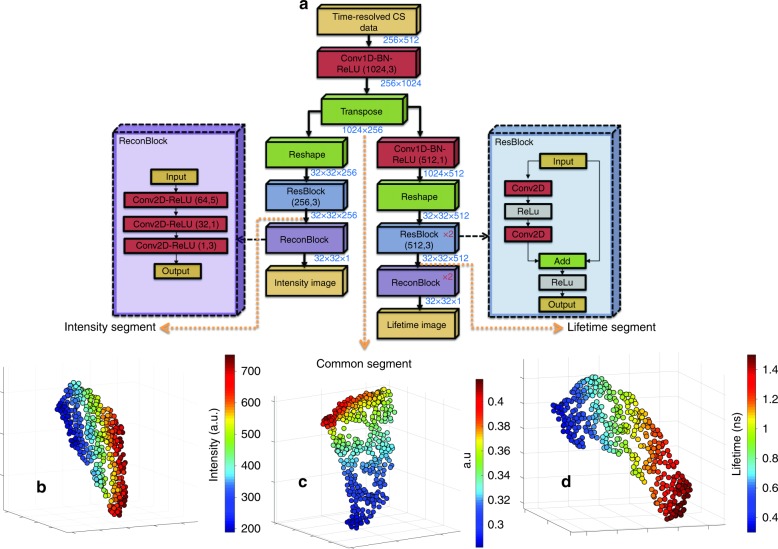
The architecture of Net-FLICS and feature maps at the end of three main segments visualized by t-SNE. **a** Net-FLICS architecture. Outputs at different endpoints of Net-FLICS as displayed in t-SNE maps for **b**. Intensity segment in photo counts **c**. Common segment, and **d** Lifetime segment in nano-seconds

Net-FLICS was implemented with Keras^18^ using TensorFlow as its backend^19^. The mean squared errors (MSE) between the reconstructed and the ground-truth intensity/lifetime images were calculated, and their sum was used as the loss function. The MSE of the lifetime was given a higher weight of 1e5 because of its smaller range compared to the intensity. RMSprop was chosen as the optimizer^20^. The learning rate was reduced by half every 10 epochs from an initial value of 1e−3. In total, 32,000 samples were used as the training data, and 8000 were used for validation. The mean absolute error (MAE) between the reconstructed and the ground-truth lifetime images was chosen as the metric to evaluate the training performance. The training was terminated when the lifetime MAE on the validation set was not improved for 10 consecutive epochs, which was 73 in this case. Fig. 2a shows the changes in MAE for intensity and lifetime reconstruction on the training and validation datasets. On a desktop with an Intel i9-7900X CPU and an NVIDIA GeForce GTX 1080 Ti GPU, each epoch took 220–225 s, which yielded a total training time of ~4.5 h. Finally, the model with the lowest lifetime validation error was used to evaluate the performance of Net-FLICS. Because of the pixels with extremely low intensity values and high Poisson noise, the final lifetime MAE of the validation set is more than twice larger than that of the training dataset. Although the intensity range is covered in our training data, the lifetime fitting for those pixels remains challenging. Meanwhile, the validation set has an even smaller final intensity MAE than the training set. The reason can be the allocation of more “harder” cases into the training set, whereas the validation set contains more “easier” cases, which also indicates no overfitting issues of Net-FLICS training. To show the outputs of Net-FLICS at distinct points in the architecture, 400 simulated samples with random intensity, lifetime and noise variations were used as the input. To display the results, maps were created from the t-distributed Stochastic Neighbor Embedding values (tSNE)^21^ calculated from the outputs after the transpose layer of the common segment in Fig. 1c and after the last ResBlock of the intensity and lifetime segments in Fig. 1b, d.

**Fig. 2:**
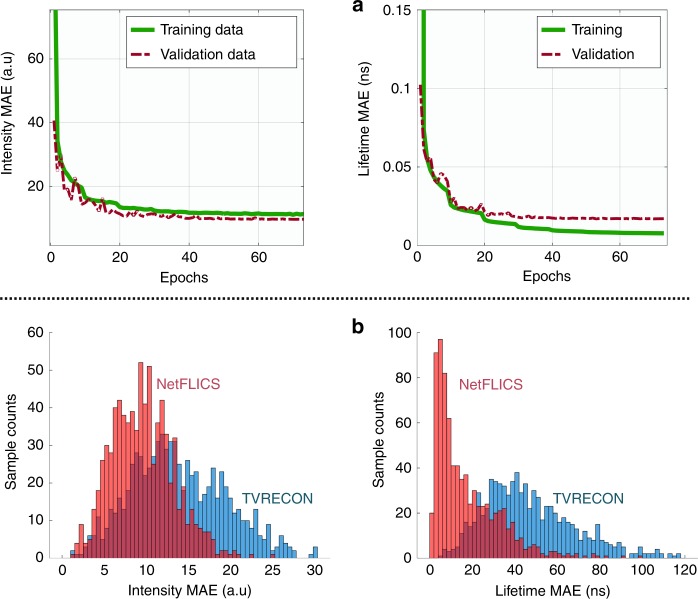
Net-FLICS MAE training curves and final MAE distributions for 800 simulated samples. **a** Intensity and lifetime MAE during training. **b** Distribution of the MAE of intensity and lifetime reconstructions for 800 simulated samples

Since tSNE propitiates dimensionality reduction, it is selected to visualize Net-FLICS data clusters by displaying the low-dimensional points. Therefore, each point of the map represents the position of a sample relative to the data cluster. The common segment displays data points that are combinations of the intensity and lifetime features. A difference is observed in the tSNE distribution of the intensity and lifetime segments, which display only intensity and lifetime features, respectively. The intensity and lifetime ranges of the common branch output are also displayed and depend on the ranges of the 400 random generated samples. Further explanation is provided in Section 5 of supplementary materials.

To demonstrate the gains of Net-FLICS, its performance was compared against TVRecon using a simulated test set with 800 samples from our data generator. The TVRecon workflow details are explained in Part 4 of supplementary materials. The trained Net-FLICS took ~2.2 s in total to reconstruct 800 samples (<3 ms/sample), whereas TVRecon yielded reconstructions using ~19.6 s per sample, which is ~7000 times longer than Net-FLICS. The distributions of the intensity and lifetime MAE for 800 reconstructions are shown in Fig. 2b. The structural similarity index (SSIM) values are also calculated between the reconstruction and the ground-truth. An MAE distribution closer to 0 indicates less variation of the lifetime reconstructions against the ground-truth, and an SSIM closer to 1 indicates higher structural similarity to the intensity ground truth. As displayed in Table 1, Net-FLICS outperforms TVRecon with lower MAE and higher SSIM for both intensity and lifetime results.

**Table 1:** MAE and SSIM of  intensity and lifetime reconstruction results on 800 simulated samples

	MAE		SSIM	
	Intensity	Lifetime	Intensity	Lifetime
TVRecon	13.80 ± 5.65	0.05 ± 0.02	0.90 ± 0.09	0.88 ± 0.09
NetFLICS	9.48 ± 3.63	0.02 ± 0.02	0.96 ± 0.05	0.95 ± 0.05

To investigate the performance of Net-FLICS on samples unknown to the data generator, in vitro experimental datasets were acquired with the proposed hyperspectral time-resolved MFLI system^3^. A phantom with RPI letters was prepared from stock solutions, which contained AF750 dye (ThermoFisher Scientific, A33085) at ~5 µM concentration for letters “R”, “I” and HITCI dye (Sigma Aldrich, 252034) at ~40 µM for letter “P”. The use of a homogeneous phantom is preferred, since MFLI focuses on retrieving the fluorescence intensities and lifetimes over macroscopic regions that typically exhibit limited variations in both quantitative parameters. Since both “R” and “I” contain AF750, they should yield similar lifetime values. This feature of the phantom serves as experimental validation. The same 512 Hadamard patterns in the data generator were used for the acquisition with exposure time of 1 s for each pattern, which yielded a total acquisition of ~17 minutes. An excitation wavelength of 740 nm and detection wavelength with the maximum fluorescence intensity at ~761 nm were used. To eliminate the effect of laser jitters, the raw experimental TPSFs were shifted, so that its 5% rising point of the 1^st^ illumination pattern (full-field) matched  those from the simulated TPSFs, which was typically implemented before the LSM-based lifetime fitting. The reconstruction results for both Net-FLICS and TVRecon are displayed in Fig. 3a. Net-FLICS reconstructions are directly quantified with no need for post-processing, whereas the background lifetime pixels are set to 0 for TVRecon. Further details on TVRecon are described in Part 4 of supplementary materials. Fig. 3b displays the distribution of values per reconstructed lifetime image. Two main distributions are observed at ~0.5 ns and ~0.9 ns, which correspond to AF750 (“R” and “I”) and HITCI (“P”). Compared to TVRecon, a smaller variation is observed for both lifetime components in Net-FLICS results, which is desired since the phantom letters are homogenous and continuous. This result can be further validated by the mean and standard deviation of lifetime reconstruction values in Table 2.

**Fig. 3:**
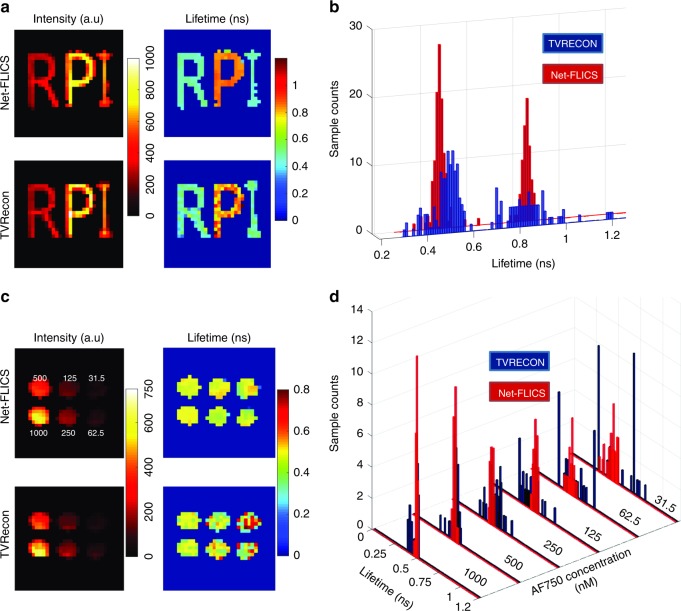
Comparison of intensity and lifetime reconstruction results between Net-FLICS and TVRecon on in vitro datasets. **a** Intensity and lifetime for RPI phantom; **b** Lifetime distribution for RPI phantom; **c** Intensity and lifetime for the decreasing AF750 concentration; **d** Lifetime distribution for the decreasing AF750 concentration

**Table 2:** Mean and standard deviation of lifetime reconstruction results on the in vitro "RPI" dataset

Lifetime (ns)	Letter “R”	Letter “P”	Letter “I”
TVRecon	0.48 ± 0.08	0.85 ± 0.11	0.49 ± 0.05
NetFLICS	0.48 ± 0.02	0.84 ± 0.07	0.47 ± 0.03

We further analyzed the performance of Net-FLICS and TVRecon under different photon count levels through simulated and in vitro experimental datasets. The simulated set contained three levels, where the maximum CW intensity of each letter/digit is distributed within 25–100 (Level 1), 100–400 (Level 2), and 400–1600 (Level 3). We used SSIM to evaluate intensity reconstructions and MAE for lifetime reconstructions. The results are displayed in Part 3 of supplementary materials. Although Net-FLICS displays a smaller-intensity SSIM for Level 1, the lifetime MAE, which is typically more important for MFLI applications, is much lower than TVRecon at all photon count levels.

The in vitro experiment contained six wells of AF750 with varying concentrations (1000 nM, 500 nM, 250 nM, 125 nM, 62.5 nM, and 31.5 nM), as displayed in Fig. 3c. This experimental design enabled us to compare the reconstruction performance of the same lifetime on a wide range of photon levels. The data processing steps are identical to those in the “RPI” phantom experiment. Since all wells contain the same dye, the distribution of the lower concentrations should ideally be similar to the distribution of the 1000 nM concentration. As shown in Fig. 3d, the outlier number in the TVRecon reconstructions significantly increases with fewer photons. In contrast, Net-FLICS yields more localized distributions at ~0.5 ns even for the lowest concentration. The mean lifetime values and standard deviations are quantified in Table 3. For both approaches, the standard deviations increase when the concentration of AF750 decreases due to higher Poisson noise. However, Net-FLICS consistently outperforms TVRecon at all photon count levels and delivers reliable results for the noisiest cases.

**Table 3:** Mean and standard deviation of lifetime reconstruction results on the in vitro wellplate dataset

nM	1000	500	250	125	62.5	31.5
TV Recon	0.46 ± 0.04	0.47 ± 0.05	0.48 ± 0.08	0.43 ± 0.10	0.47 ± 0.15	0.53 ± 0.18
Net FLICS	0.50 ± 0.01	0.48 ± 0.02	0.45 ± 0.02	0.50 ± 0.02	0.46 ± 0.04	0.47 ± 0.06

Finally, we tested the performance of Net-FLICS using an in vivo mouse dataset^3^. The mouse was injected with transferrin-conjugated AF700 and AF750, and the measurements were taken 4 h and 6 h post injection with the single-pixel system^3^ to analyze the FRET interaction inside the liver and urinary bladder over time. Measurements at the emission wavelength with the highest intensity (730 nm) were selected. Unlike the above in vitro experiments, only the first 400 Hadamard pattern pairs, ranked by spatial frequency, were used to achieve shorter data acquisition time^22^. Shorter acquisition times are desired due to animal handling protocols, so the pattern number was reduced. The size of the first Conv1D layer of Net-FLICS varied, but all other structures remained the same. The mean and standard deviation of lifetime values for both organs from two approaches are displayed in Table 4. In such scenarios, it is not possible to obtain a ground truth, so we are limited in reporting the descriptive statistics though reported values are in agreement with previousy published results^3^. As displayed in Fig. 4 and in accordance with the in vitro experiments, the reconstructions from Net-FLICS obtain a smaller variation than TVRecon. The smaller standard deviations for Net-FLICS result in smoother FRET mean lifetime maps, which are expected for in vivo conditions. Indeed, as these measurements were obtained from intact live animals, the fluorescence maps acquired on the animal surface are diffused by nature and consequently should demonstrate smooth features in both intensity and FLI.

**Table 4:** Mean and standard deviation of lifetime reconstruction results on the in vivo mouse dataset

Lifetime (ns)	Liver (4 h)	Bladder (4 h)	Liver (6 h)	Bladder (6 h)
TVRecon	0.75 ± 0.17	0.90 ± 0.10	0.64 ± 0.26	0.93 ± 0.08
NetFLICS	0.77 ± 0.12	0.88 ± 0.10	0.58 ± 0.17	0.89 ± 0.10

In summary, we reported a new CNN named Net-FLICS, which enables us to generate quantitative fluorescence intensity maps and FLI images at computational speeds 4 orders of magnitude faster than the current inverse-based and fitting methodologies (~7000 times our computational platform). This novel network design was trained and extensively validated using a model-generated simulation dataset that removed the need to train with experimental data. Moreover, it was also validated with independent experimental datasets that were not from the data generator. In all cases, both in vitro and in vivo, Net-FLICS produced quantitative intensity and FLI maps with improved accuracy than the classical TVRecon approach previously used.

**Fig. 4:**
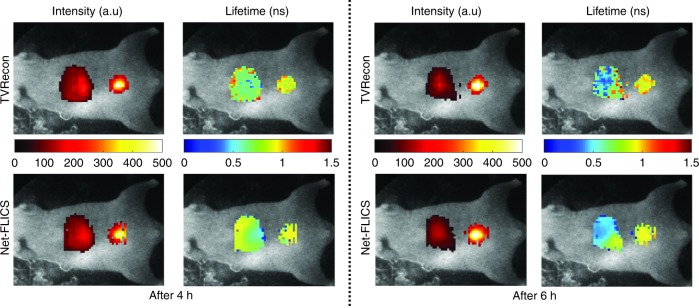
**In vivo intensity and mean lifetime reconstructions at 4 hours and 6 hours post-injection**

Of importance to numerous bio-photonics applications, the FLI quantification provided by Net-FLICS was superior under photon-starved conditions. Hence, we expect that Net-FLICS and other deep learning methodologies will further cement the utility of lifetime-based parameters for biomedical applications. Furthermore, Net-FLICS was trained via a model-based approach but still provided outstanding results with experimental data without requiring a tweaking of network parameters. Hence, Net-FLICS is a fitting-free and inverse solver-free user-friendly methodology that could improve the acceptance of FLI by non-expert user communities such as biologists, drug development scientists, and surgeons.

## Supplementary information

Supplemental Information
