# Supplementary material for: Net-FLICS: fast quantitative wide-field fluorescence lifetime imaging with compressed sensing – a deep learning approach
Source: Light Sci Appl. 2019 Mar 6;8:26. doi: 10.1038/s41377-019-0138-x (PMC6400960; doi:10.1038/s41377-019-0138-x)
Supplement: Supplementary file 1 — Supplemental Information [file 41377_2019_138_MOESM1_ESM.docx]

Supplementary Information

for

**Net-FLICS: Fast Quantitative Wide-field**

**Fluorescence Lifetime Imaging with**

**Compressed Sensing – A deep learning**

**approach**

Ruoyang Yao, Marien Ochoa, Pingkun Yan and Xavier Intes

e-mail: [xintes@rpi.edu](mailto:xintes@rpi.edu) ,

[yaoruoyang@gmail.com](mailto:yaoruoyang@gmail.com)

1. Full details of Net-FLICS architecture

The proposed Net-FLICS takes in a 2D array of size 256×512, representing 512 CS measurements, each with 256 time gates, as displayed in **Fig. S1**, and outputs an intensity image and a lifetime image, both of size 32×32, as the reconstruction predictions.

**Why Net-FLICS is designed in this way:**

Net-FLICS aims to reconstruct both intensity and lifetime images from single-pixel compressed sensing data. Since ReconNet^1^ is an established architecture for recovering images from compressed sensing data with robust performance when compared to state-of-the-art solvers, it is a logical foundation for our application. However, ReconNet takes compressed sensing images as input, while Net-FLICS takes a more challenging input array of 256×512, corresponding to 512 CS measurements in the time domain through 256 time gates. Therefore in this paper, we propose a new network with three-segment structure.

The first segment of Net-FLICS is dedicated to expanding from 1D CW data to 2D temporal data compared to the first layer of ReconNet. However, given the higher complexity of time-resolved data and lifetime fitting, more processing is required before the ReconBlocks can be applied to retrieve the final intensity and lifetime reconstructions. We started by adding some simple convolutional layers but the results were not satisfying because a lot of times the training errors stopped decreasing after 1-2 epochs. Such results suggested that the initial model was not powerful enough to process the input data and indicated the need for a deeper network. Therefore, we decided to adopt the ResNet design, which can effectively address the vanished gradient issue when building very deep and powerful networks. The results were then significantly improved. As shown in **Fig. 1** of the manuscript, the intrinsic manifold structure of the data can be well recovered after the first segment with ResNet structure. From that point on, we utilized two ReconNet structures as segments 2 and 3 for lifetime and intensity reconstructions. Simultaneous reconstruction is conducted in the spirit of multi-task learning to achieve good final reconstruction performance.

**Full details of Net-FLICS architecture:**

There are three main segments in Net-FLICS as illustrated in **Fig. 1(a)**, one shared by both reconstruction tasks, followed by two branches devoted to intensity and lifetime image restoration respectively.

**The shared segment** aims to recover sparsity information from CS data, similar to the first step in the TVRecon workflow^2,3^. Since the input of Net-FLICS has one more dimension than that of ReconNet, we replace the fully-connected layer with a 1D convolutional layer. The layer contains 1024 1D convolutional kernels of size 3 by 512 (number of patterns). Each kernel would slide through the input signal along the temporal dimension and lead to an output signal of size 256 by 1. The length of the kernel window is set to 3 so that the raw time-resolved measurements with Poisson noise can be smoothened by averaging data from neighboring gates. The output array from the first segment (256×1024) is transposed so each row represents the Temporal Point Spread Function (TPSF) of one pixel in the 32×32 image. In the branch of intensity reconstruction (**Segment 2**), the output array from Segment 1 is first reshaped into 32×32×256, and then processed with one ResBlock and one ReconBlock to generate the final intensity image. The three 2D convolutional layers in the ReconBlock has the same number of feature maps (kernels) as in the original ReconNet paper, but the kernel sizes are modified to accommodate our own application, as labeled in **Fig. 1**.

Since the reconstruction of lifetime image is more involved than that of intensity, the corresponding branch (**Segment 3**) in Net-FLICS is also more complex. After the transpose operation at the end of Segment 1, the feature axis of data has changed from pattern to time gate. We first increase the number of features from 256 to 512 through a 1D convolutional layer, where the kernel size is set as 1 because the adjacent pixels in the current 2D array are not necessarily adjacent in the real world. After the data is reshaped to 32×32×512, it is then processed with two ResBlocks and two ReconBlocks to generate the final lifetime image. The numbers of kernels are doubled in the first ReconBlock due to the increased number of features in the lifetime data.

The commonly applied rectified linear unit (ReLU)^4^ is adopted as the activation function throughout the design of Net-FLICS. In addition, due to the large range of intensity value distribution between different patterns, time gates and samples, Batch Normalization (BN)^5^ is applied after all 1D convolutional operations and before ReLU activations to eliminate possible saturating issues and speed up the convergence of training. However, BN is not applied for 2D convolutional layers, as what has been implemented in the ReconNet paper.

**2. Data Generator for Net-FLICS training**

We took advantage of the EMNIST^6^ dataset, which was originally created for handwriting recognition research purpose , as the raw input of the data generator. The complete EMNIST training dataset contains ~7×10^5^ grayscale images of size 28×28, each representing a digit or a letter (uppercase/ lowercase). To start with, the letter or digit was resized to fully occupy the 28×28 space and padded with 0s equally on four sides to obtain a 32×32 image $\boldsymbol{I}_{0}$. We then converted each of them to a binary image ***I***, with a threshold of 0.5**max*($\boldsymbol{I}$).

Next, for all foreground pixels in the image $I_{i,j}$, where $1\leq i, j\leq32$ are the row and column index, a uniform continuous wave (CW) intensity value $A$ was randomly drawn in the range from 25 to 1600 and a uniform lifetime value $\tau$ was generated between 0.3 and 1.5 ns. We then applied a Gaussian filter to the intensity image to generate smooth boundary but kept the lifetime constant at all foreground pixels.

For data augmentation purpose, each sample contains 1×1, 1×2, …, 1×4, 2×1, …, 4×4 letters/digits. Intensity images $\boldsymbol{I}_{A0}$ and lifetime images $\boldsymbol{I}_{\tau0}$ of size 32×32 are stitched together and resized back to a 32×32 image. For example, as shown in the **Fig. S2**, the second row is a sample containing 3×2 digits/letters. It’s generated by stitching 6 images of size 32×32 to an image of size of 96×64 and then reshaped to 32×32 through down-sampling. Similarly, the third row is a sample containing 1×4 digits/letters. To avoid possible patterns in the training dataset, each letter/digit is randomly flipped and rotated by a multiple of 90 degrees. These were the final intensity and lifetime images used as the ground truth for the output of Net-FLICS.

With the intensity, lifetime, time gate width of the imaging system (in our case, 32.6 ps per gate and 256 gates in total), we were able to generate an intensity decay curve for each pixel. The curve was then convoluted with the instrumental response function (IRF) to obtain TPSF. Single-pixel measurements were calculated as the weighted sum of TPSFs from all pixels in the image, where the weights ranging [-1, 1] come from the illumination/detection patterns used for data acquisition. In our experiment, 512 out of 1024 Hadamard patterns ranked by increasing spatial frequency, each containing 32×32 pixels, were adopted as illumination patterns, while a full-field uniform pattern was used for detection^3,7^. This led to a compression ratio (CR) of 50% but other CR values could other be explored. Since Hadamard patterns contain both positive and negative values, each pattern was decomposed to a complementary pair, leading to a total of 1024 measurements. Finally, time-resolved single-pixel data were added with Poisson noise, subtracted pairwise and used as the input for Net-FLICS, which was a 2-D matrix of size 256×512.

3. Net-FLICS performance under different photon count levels

Results for the simulated EMNIST data sets at three different photon count levels are displayed in **Fig. S3.** The maximum CW intensity of each letter or digit is distributed by photon counts (p.c) in Level 1 with 25 to 100 p.c, Level 2 with 100 to 400 p.c and Level 3 with 400 to 1600 p.c. SSIM is used to evaluate intensity reconstructions and MAE for lifetime reconstructions. Although Net-FLICS displays smaller intensity SSIM for Level 1, the lifetime MAE, typically more important for MFLI applications, is much lower than TVRecon at all photon counts levels. Especially, the average lifetime MAE of Net-FLICS for Level 1 (0.039 ns) is almost the same as that of TVRecon for Level 3 (0.038 ns). These results are further specified on **Table S1**.

**Table S1. Intensity SSIM and lifetime MAE per photon count level.**

| P.C. Level | 25-100 Maximum Photons | | 100-400 Maximum Photons | | 400-1600 Maximum Photons | |
| --- | --- | --- | --- | --- | --- | --- |
| Metrics | **Intensity SSIM** | **Lifetime MAE** | **Intensity SSIM** | **Lifetime MAE** | **Intensity SSIM** | **Lifetime MAE** |
| TVRecon | 0.85 ± 0.09 | 0.10 ± 0.02 | 0.89 ± 0.09 | 0.06 ± 0.02 | 0.91 ± 0.08 | 0.04 ± 0.01 |
| Net-FLICS | 0.78 ± 0.10 | 0.04 ± 0.02 | 0.97 ± 0.02 | 0.01 ± 0.01 | 0.98 ± 0.03 | 0.01 ± 0.01 |

**4. Workflow of TVRECON**

In the TVRecon workflow, CW intensity images were first reconstructed using TVAL3^2^, a state-of-art inverse solver for Compressed Sensing [20]. TVAL3 is a parameter dependent algorithm, which by default makes Net-FLICS a more direct approach. The main parameters were defined by tuning values suggested by the algorithm developers^8^ until obtaining reconstructions with highest SSIM value. An *anisotropic* and nonnegative model with the following main parameters was specified:

primary penalty parameter *µ* = 2e8,

secondary penalty parameter *β* = 2e13,

outer stopping tolerance 𝜀 = 1e-3,

maximum total iterations = 300.

As described in paragraph 1 of the manuscript, TVAL3 seeks to inverse solve the image sample plane, resulting in a 32×32×256 array which represents 32×32 images over 256 time points. Therefore, each of the pixels in the 32×32 space contains a Time Point Spread Function (TPSF) with photo-counts that correspond to the intensity of that respective pixel and a fluorescent decay corresponding to the lifetime of the fluorophore. This time-domain (TD) data can be: (1) Integrated over the time points dimension to construct a Continuous Wave (CW) intensity image and (2) fitted through the time dimension using a constrained Least-Squares based minimization algorithm for three lifetime (*τ*) ranges: [0.3 0.7] ns, [0.7 1.1] ns and [1.1 1.5] ns. The lifetime with the lowest fitting residual was then used as the pixel lifetime (*τ*) value. Please see Refs. 2 and 6 for further details. To reduce processing time the lifetime fitting is only applied to a defined Region of Interest (ROI) which is calculated by intensity thresholding. Only values that are within 5% for simulated and 20% for more noisy experimental datasets of the maximum CW intensity are used. This assures only TPSFs with enough intensity are quantified.

**5. tSNE Maps**

t-distributed Stochastic Neighbor Embedding (tSNE) is an algorithm that has been proposed for visualizing high-dimensional data. In the case of Net-FLICS, data is composed of both intensity and lifetime features per measured pattern and sample, therefore in order to easily display how the data looks like at different points within the network a dimensionality reduction method is desired. Three points within Net-FLICS were chosen for tSNE map calculations: one in the common segment where data contains intensity and lifetime features, the second and third maps describe data at the intensity and lifetime branches respectively. To generate the tSNE maps Net-FLICS model and activation layers were applied to 400 simulated validation samples. Each map contains the low-dimensional analogues of the high-dimensional data points at a specific branch of the network.

**References**

1 Kulkarni, K., Lohit, S., Turaga, P., Kerviche, R. & Ashok, A. in *2016 IEEE Conference on Computer Vision and Pattern Recognition (CVPR).* 449-458.

2 Li, C. Compressive sensing for 3D data processing tasks: applications, models and algorithms. (2011).

3 Ochoa, M., Pian, Q., Yao, R., Ducros, N. & Intes, X. Assessing patterns for compressive fluorescence lifetime imaging. *Optics Letters* **43**, 4370-4373 (2018).

4 Nair, V. & Hinton, G. E. in *Proceedings of the 27th international conference on machine learning (ICML-10).* 807-814.

5 Ioffe, S. & Szegedy, C. Batch normalization: Accelerating deep network training by reducing internal covariate shift. *arXiv preprint arXiv:1502.03167* (2015).

6 Cohen, G., Afshar, S., Tapson, J. & van Schaik, A. EMNIST: an extension of MNIST to handwritten letters. *arXiv preprint arXiv:1702.05373* (2017).

7 Pian, Q., Yao, R., Sinsuebphon, N. & Intes, X. Compressive hyperspectral time-resolved wide-field fluorescence lifetime imaging. *Nature photonics* **11**, 411 (2017).

8 Li, C., Yin, W. & Zhang, Y. User’s guide for TVAL3: TV minimization by augmented lagrangian and alternating direction algorithms.

**Figure Captions**


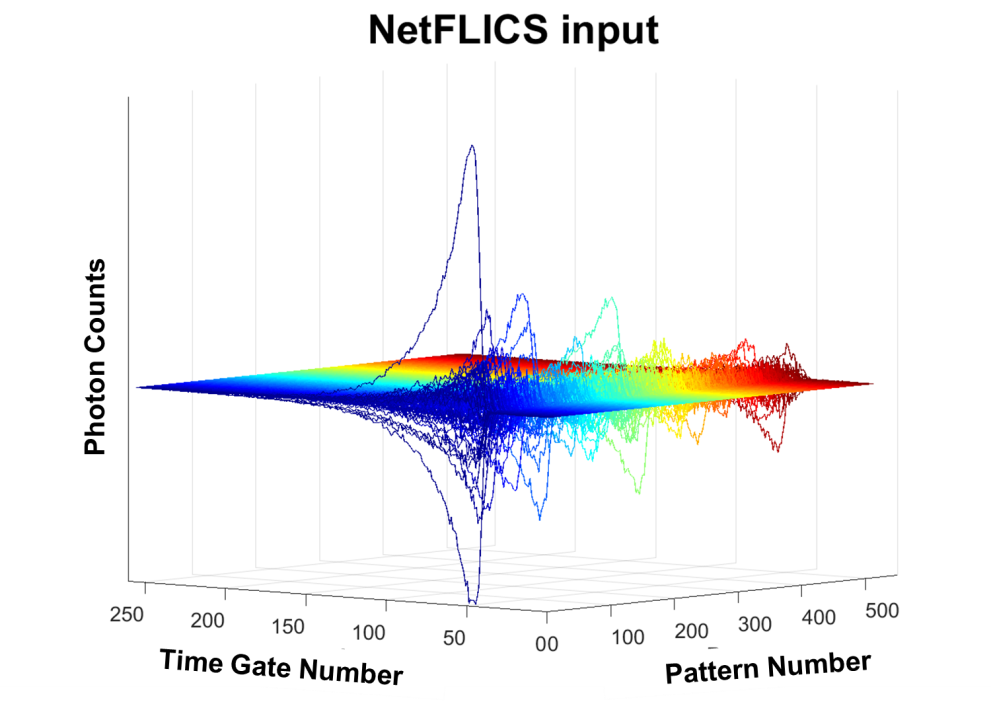


**Fig. S1.** Example of Net-FLICS input data with dimensions 256×512.


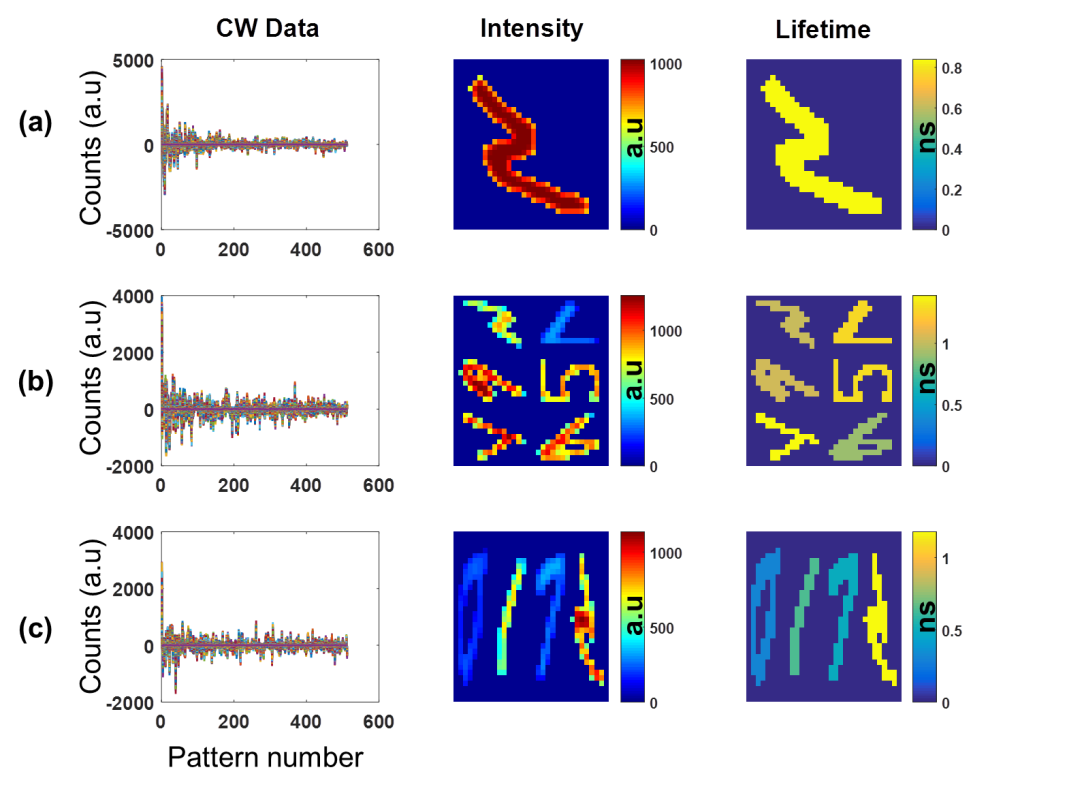


**Fig. S2.** EMNIST example dataset. Generated continuous wave (CW) data (averaged over 256 time channels), intensity and lifetime images for samples (a), (b) and (c).

**
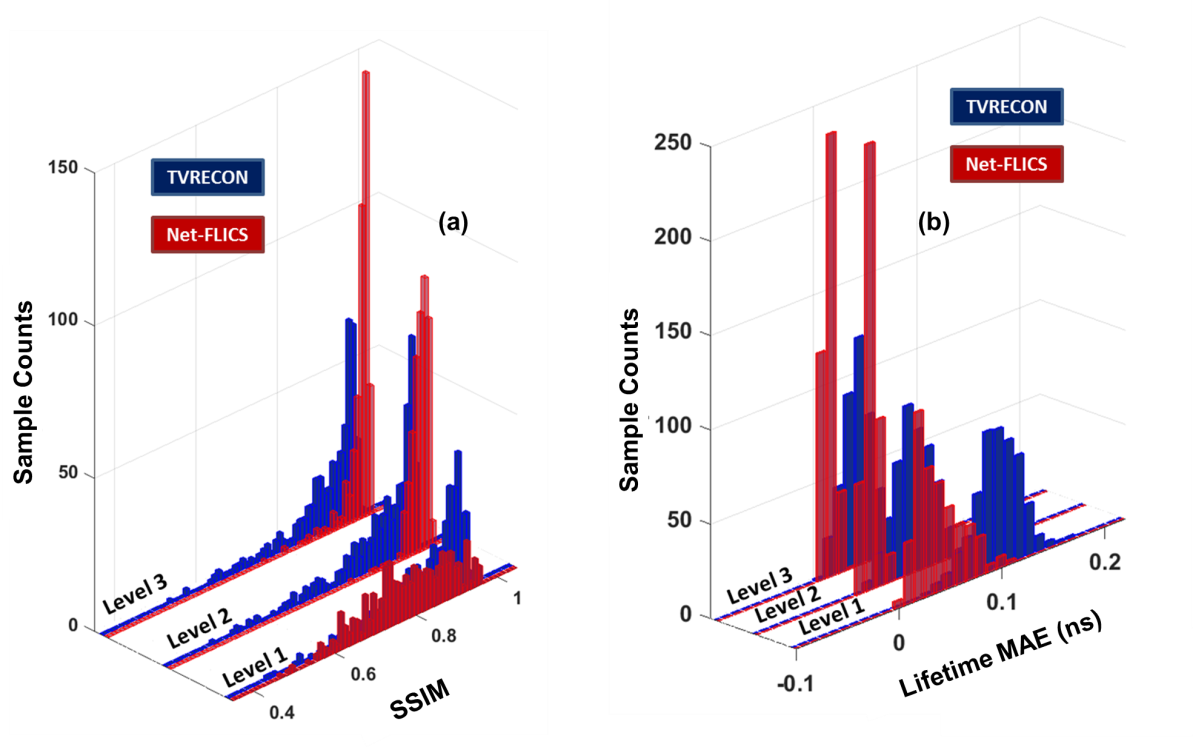
**

**Fig. S3.** Results for photon count levels 1-3. (a) Intensity SSIM (b)Lifetime MAE.
